# *Tcf12* Is Involved in Early Cell-Fate Determination and Subset Specification of Midbrain Dopamine Neurons

**DOI:** 10.3389/fnmol.2017.00353

**Published:** 2017-11-01

**Authors:** Simone Mesman, Marten P. Smidt

**Affiliations:** Swammerdam Institute for Life Sciences, FNWI University of Amsterdam, Amsterdam, Netherlands

**Keywords:** *Tcf12*, dopamine neuron, midbrain development, bHLH factors, early cell-fate commitment, subset specification

## Abstract

The basic helix-loop-helix (bHLH) protein family has previously been shown to be involved in the development of mesodiencephalic dopaminergic (mdDA) neurons in the murine midbrain. Specifically, *Ngn2* and *Mash1* are known to have a role in the specification of neural progenitors in the ventricular zone (VZ) of the midbrain towards an mdDA neuronal cell-fate. Furthermore, other members of the bHLH protein family, the E-box factors, are expressed in the developing midbrain and are thought to have a role in neuronal differentiation. Here we show that the E-box factor *Tcf12* is implicated in early and late development of mdDA neurons. *Tcf12* is expressed in the midbrain and in young TH-expressing mdDA neurons throughout development. *Tcf12*^lox/lox^;*En1*^cre/+^ embryos, that lose *Tcf12* at ~embryonic day (E)9 throughout the *En1* expression domain, have a changed spatial expression of *Lmx1a* and *Nurr1* and a consistent loss of rostral TH expression. Expression of the subset marker *Ahd2* is initially delayed, but recovers during development, eventually showing an ~10% increase in AHD2-expressing cells at postnatal day (P)30. *Tcf12*^lox/lox^;*Pitx3*^cre/+^ embryos, that lose *Tcf12* at ~E12 in post-mitotic mdDA neurons, show no effect on the amount of TH-expressing neurons in the developing midbrain. However, similar as to *Tcf12*^lox/lox^;*En1*^cre/+^ embryos, subset specification is delayed during development. Taken together, we have identified *Tcf12* as a novel factor in mdDA neuronal development. It serves a dual function; one in early cell-fate commitment of neural progenitors and one late in subset specification.

## Introduction

The mesodiencephalic dopaminergic (mdDA) system is involved in motor control, reward and motivation in the adult brain. Neurons that make up the mdDA system are thought to arise from neural progenitors present in the ventricular zone (VZ) of the floor plate (FP) and basal plate (BP) of the embryonic midbrain (Ono et al., 2007; Mesman et al., 2014). After birth mdDA neurons migrate towards their final location and acquire a subset-specific identity (Di Salvio et al., 2010; Jacobs et al., 2011; Hoekstra et al., 2013; Smits et al., 2013; Veenvliet et al., 2013), of which the neuroanatomically distinct substantia nigra (SNc) and ventral tegmental area (VTA) are best known, also due to the selective degeneration of the SNc during Parkinson’s Disease (PD; Barzilai and Melamed, 2003).

Signaling molecules, like members of the WNT-family and FGF8, are shown to be important for neural progenitors to acquire an mdDA neuronal fate, and early specification factors, like *Lmx1a*, *Lmx1b*, *Nurr1* and *En1*, are known to be crucial to acquire the correct (subset) identity (Smits et al., 2006; Abeliovich and Hammond, 2007; Smidt and Burbach, 2007; Roybon et al., 2008; Allodi and Hedlund, 2014). One specific protein family that is involved in neuronal development is the basic helix-loop-helix (bHLH) protein family (Powell and Jarman, 2008). The bHLH protein family consists of transcription factors with a bHLH domain which allows them to bind DNA by the formation of homo- and heterodimers with other bHLH proteins (Murre et al., 1989; Powell and Jarman, 2008). The function of bHLH proteins depends on their interaction partner and spatio-temporal expression pattern (Powell and Jarman, 2008). Two members of this family, *Ngn2* and *Mash1*, have been show to have a role in the onset of mdDA neuronal development (Andersson et al., 2006a; Kele et al., 2006). Deletion of *Ngn2* leads to a delay of mdDA neuronal development, eventually resulting in an underdeveloped mdDA neuronal population with a loss of TH-expressing mdDA neurons in both VTA and SNc (Andersson et al., 2006a; Kele et al., 2006). This phenotype is partially compensated by its family-member* Mash1* (Kele et al., 2006), indicating that bHLH proteins function together in mdDA neuronal development.

The bHLH protein family is an enormous family that can be subdivided into different smaller subfamilies (Skinner et al., 2010). One subfamily is the E-box protein family that consists out of three different genes: *Tcf12* (*Heb*), *Tcf3* (*E2A*) and *Tcf4* (*E2-2*, Massari and Murre, 2000). Although these proteins have a broad expression pattern throughout the embryo, most research has been performed to their function in immune system development. E-box proteins have been implicated in differentiation and proliferation during T-cell development. They are important regulators in the coordination of differentiation and proliferation events in the pre-mature T-cell, before further development and fate-commitment (Wojciechowski et al., 2007). *Tcf12* regulates the transition of CD4^−^CD8^−^ double-negative precursor cells to the CD4^+^CD8^+^ double-positive stage of development, whereas *Tcf3* inhibits the transition from the CD4^+^CD8^+^ double-positive to the CD4^+^ or CD8^+^ single-positive stage of development, which eventually become part of the different subsets of T-cells present in the immune system (Wojciechowski et al., 2007). In general, loss of one of these factors results in an early depletion of T-cell progenitors and a decrease of mature T-cells. *Tcf4* on the other hand has only a minor role in thymocyte development and loss of this gene does not lead to gross effects on the T-cell population (Wojciechowski et al., 2007; Wikström et al., 2008).

During brain development, E-box proteins are thought to regulate common targets for neurogenesis and unique target genes for neuronal subtype development, dependent on their interaction partner and area of expression (Powell and Jarman, 2008). Although a complete loss of *Tcf12* and *Tcf3* leads to an overall reduction in brain size, the morphology of the brain is preserved in these mutants (Ravanpay and Olson, 2008). Other studies have implied that loss of *Tcf12* leads to exencephaly and that, at least in the cortex, *Tcf12* is likely involved in sustaining the undifferentiated state of proliferating progenitor cells during neurogenesis (Barndt et al., 2000; Uittenbogaard and Chiaramello, 2002). Loss of *Tcf4* was shown to have a relative mild effect on development of a region in the pons (Flora et al., 2007). However, haplo-insufficiency of *Tcf4* in humans leads to Pitt-Hopkins Syndrome (Flora et al., 2007; Sweatt, 2013), which is characterized by major defects in cortical development.

In this study, we show that both *Tcf12* transcript and TCF12 protein are present in the VZ of the embryonic mesodiencephalic FP and co-localize with young TH-expressing neurons medial in the TH^+^ neuronal population. We investigated the role of *Tcf12* in mdDA neuronal development, by means of conditional deletion of *Tcf12* in the midbrain via use of two cre-drivers; the knock-out knock-in *En1*^cre^ driver for early deletion (~E9) of *Tcf12* throughout the *En1* expression area (Kimmel et al., 2000), and the knock-out knock-in *Pitx3*^cre^ driver for late deletion (~E12) of *Tcf12* within post-mitotic mdDA neurons (Smidt et al., 2012). *En1*^cre^ driven deletion of *Tcf12* affects the expression of the early mdDA neuronal specification factors *Lmx1a* and *Nurr1*, but has no effect on the expression of *Ngn2*. We detected a loss of mdDA neurons and affected subset specification in the *Tcf12*^lox/lox^; *En1*^cre/+^ mutants, which continues into adult stages. *Pitx3*^cre^ driven loss of *Tcf12*, on the other hand, has no effect on the TH-expressing neuronal population, but does result in a developmental delay of mdDA neuronal subset specification.

Taken together, our data show that the E-box protein *Tcf12* is a novel factor involved in the development and specification of mdDA neurons. At early stages of development, it plays an important role in the onset of mdDA neuronal specification and development of TH-expressing neurons, whereas at late stages, it is involved in the start of subset specification and correct timing of expression.

## Materials and Methods

### Ethics Statement

All animal studies were performed in accordance with local animal welfare regulations, as this project has been approved by the animal experimental committee (Dier ethische commissie, Universiteit van Amsterdam, DEC-UvA), and international guidelines.

### Animals

The *Tcf12^flox^* (*Heb^flox^*) mice strain was a kind gift of Prof Dr. Y. Zhuang from the Department of Immunology of the Duke University Medical Center in Durham, North Carolina (Wojciechowski et al., 2007). Embryos were generated by crossing with *En1^cre^* (Kimmel et al., 2000) and *Pitx3^cre^* (Smidt et al., 2012) mice strains. WT embryos, to investigate normal expression of *Tcf12*, were generated by crossing C57BL/6 mice. Pregnant (embryonic day 0.5 (E0.5) is defined as the morning of plug formation) and adult mice were sacrificed by cervical dislocation. Embryos and brains were collected in 1× PBS and immediately frozen on dry-ice, or fixed by immersion for 3–12 h in 4% paraformaldehyde (PFA) at 4°C. After PFA incubation, samples were washed in 1× PBS and cryoprotected O/N in 30% sucrose at 4°C. Embryos and brains were frozen on dry-ice and stored at −80°C. Cryosections were sliced at 16 μm, mounted on Superfrost Plus slides (Thermo Fisher Scientific), air-dried, and stored at −80°C until further use.

### Genotyping

Genotyping of the *Tcf12^flox^* allele was performed as described (Wojciechowski et al., 2007). PCR was performed with 50 ng of genomic DNA together with FP 5′-CTGGGACAGAAGTTCAGCACTTAGTAC-3′ and RP 5′-CATTCCTATACATCAGCTTCTTGGACG-3′ resulting in a product at 1.1 kb for the WT allele and a product at 1.3 kb for the floxed allele (Supplementary Figure S1A).

Genotyping of the *En1^cre^* allele was performed with 50 ng of genomic DNA together with primer pair En1Cre 5UTR_F3 5′-CTTCGCTGAGGCTTCGCTTT-3′ and En1Cre Cre_R2 5′-AGTTTTTACTGCCAGACCGC-3′ resulting in a product at 240 bp for the cre-allele (Supplementary Figure S1B).

Genotyping of the *Pitx3^cre^* allele was performed as described (Smidt et al., 2012). PCR is performed in two reactions. The first reaction contained 50 ng of genomic DNA together with primer pair iCre-FP2 5′-GCATGATTTCAGGGATGGAC-3′ and iCre-RP2 5′-ATGCTCCTGTCTGTGTGCAG-3′ resulting in a product at 750 bp for the cre-allele. The second reaction contained 50 ng of genomic DNA together with primer pair Pitx3-exon2–3_FP 5′-CAAGGGGCAGGAGCACA-3′ and Pitx3-exon2-3_RP 5′-GTGAGGTTGGTCCACACCG-3′ resulting in a product at 400 bp for the WT allele (Supplementary Figure S1C).

### *In Situ* Hybridization and Combined TH-DAB IHC

*In situ* hybridization with digoxigenin (DIG)-labeled probes was performed as described previously (Smidt et al., 2004). Fresh frozen sections were fixed in 4% PFA for 30 min and acetylated with 0.25% acetic anhydride in 0.1 M triethanolamine for 10 min. Probe hybridization was performed at 68°C O/N with a probe concentration of 0.4 ng/μl in a hybridization solution containing 50% deionized formamide, 5× SSC, 5× Denhardt’s solution, 250 μg/ml tRNA Baker’s yeast, and 500 μg/ml sonificated salmon sperm DNA. The following day slides were washed in 0.2 × SSC for 2 h at 68°C followed by blocking with 10% HIFCS in buffer 1 (100 mM TricHCl, pH = 7.4 and 150 mM NaCl) for 1 h at RT. DIG-labeled probes were detected by incubating with alkalin-phosphatase-labeled anti-DIG antibody (Roche, Mannheim), using NBT/BCIP as a substrate. DIG *in situ* hybridization was performed with the following probes: 965 bp *Tcf12* fragment bp 1027–1992 of mouse cDNA, 491 bp *Th* fragment 252–743 of mouse cDNA, 359 bp *Dat* fragment bp 762–1127 of rat cDNA, 1796 bp *Ahd2* fragment bp 5–1801 of mouse cDNA, 368 bp *Cck* fragment bp 263–630 of mouse cDNA, 1150 bp fragment *Lmx1a* bp 218–1366 of mouse cDNA, 2246 bp *Nurr1* fragment bp 1–2247 of rat cDNA, and 707 bp *Ngn2* fragment bp 98–1002 of mouse cDNA. Slides were washed 2 × 5 min in T_10_E_5_, dehydrated with ethanol, and embedded in Entellan.

After DIG *in situ* hybridization for *Tcf12*, E12.5, E14.5, and adult WT sections were immunostained for TH. Slides were incubated in 0.3% H_2_O_2_ in Tris-buffered saline (TBS) for 30 min at RT. Thereafter, blocking was performed with 4% heat-inactivated fetal calf serum (HIFCS) in TBS. Slides were incubated O/N with primary antibody Rb-TH (Pelfreeze, 1:1000) in TBS. The following day slides were incubated for 1 h with biotinylated goat-anti-rabbit and 1 h with avidin-biotin-peroxidase reagents (ABC Elite kit, Vector Laboratories 1:1000) in TBS. Slides were stained with 3,3′-diamino-benzidine (DAB) for a maximum of 10 min. Slides were dehydrated with ethanol and embedded with Entellan.

### Immunohistochemistry

Fluorescence immunohistochemistry was performed as described previously (Kolk et al., 2009; Fenstermaker et al., 2010). Cryosections were blocked with 4% HIFCS or 5% normal donkey serum (for sheep primary antibodies) in 1× THZT and incubated O/N with a primary antibody (Rb-TH (Pelfreeze 1:1000), Sh-TH (Millipore AB1542, 1:1000), Rb-PITX3 ((Smidt et al., 2000) 1:1000), Rb-TCF12 (A-20, Santa Cruz sc-357, 1:250), Rb-Ki67 (Abcam ab15580, 1:500), Rb-PhosphoH3 (Abcam ab5176, 1:1000)). The next day sections were incubated with a secondary Alexafluor antibody (anti-rabbit, anti-sheep) diluted 1:1000 in 1× TBS for 2 h at RT. After washing with 1× PBS, slides were incubated with DAPI (1:3000) 5 min at RT and after extensive washing with 1× PBS embedded in Fluorsave (Calbiogen). Analysis was performed on a fluorescent microscope.

DAB immunohistochemistry on adult sections was performed with Rb-AHD2 (Abcam ab23375 1:1000) as described above, with some alterations. The AHD2 antibody required antigen retrieval as follows. Cryosections were incubated with 0.1 M citrate buffer pH6 for 3 min at 800 W and 9 min a 400 W, cooled down to RT in a water bath. Thereafter slides were blocked in 4% HIFCS in 1× THZT and incubated O/N with a primary antibody. The following day slides were incubated for 1 h with biotinylated goat-anti-rabbit and 1 h with avidin-biotin-peroxidase reagents (ABC Elite kit, Vector Laboratories 1:1000) in TBS. The slides were stained with DAB for a maximum of 10 min. Dehydration was performed with ethanol and embedding with Entellan.

### Quantification of Immunohistochemistry

Quantification of TH-, PITX3- and P-H3-expressing cells was performed in ImageJ as follows. TH- and PITX3-expressing cells were counted in 5–10 (matching) sagittal sections of E12.5 and E14.5 embryos (*n* = 3 *Tcf12^+/+^*;*En1^cre/+^*/*Tcf12^+/+^*; *Pitx3^cre/+^* *n* = 3 *Tcf12^lox/lox^*;*En1^cre/+^*/*Tcf12^lox/lox^*;*Pitx3^cre/+^*). P-H3 expressing cells were counted in 5 (matching) coronal sections of E12.5 embryos (*n* = 3 *Tcf12^+/+^*;*En1^cre/+^* *n* = 3 *Tcf12^lox/lox^*;*En1^cre/+^*). Cells were counted as positive, if staining co-localized with a nuclear DAPI staining.

TH- and AHD2-expressing cells in the adult brain were quantified in 6–11 (matching) coronal sections of P30 adult brains (*n* = 3 *Tcf12^+/+^*;*En1^cre/+^*/*Tcf12^+/+^*;*Pitx3^cre/+^* *n* = 3 *Tcf12^lox/lox^*;*En1^cre/+^*/*Tcf12^lox/lox^*;*Pitx3^cre/+^*). The SNc and VTA were determined based on anatomical landmarks for quantification of the TH-expressing cells. The SNc was clearly determined in sections rostral from the fasciculus retroflexus, whereas the mdDA neuronal population was divided in the VTA and SNc in sections containing and caudal from the fasciculus retroflexus. The distinction between the SNc and VTA was made based on the tracts of the medial lemniscus, positioned in between the SNc and VTA. TH-expressing cells were counted as positive when staining co-localized with nuclear DAPI staining. Quantification of AHD2^+^ cells performed on DAB-immunohistochemistry cells were counted as positive when they showed specific DAB staining in the cytoplasm.

Quantifications are expressed as percentages of the WT (with WT set at 100%) ± standard deviation. Parametric statistical analysis was performed through a student’s *t*-test. *p* < 0.05 was considered significant, and indicated using an asterisk (*).

## Results

### *Tcf12* Is Expressed in the Embryonic Midbrain throughout Development, and Co-Localizes with Young TH-Expressing Neurons

**Figure 1:**
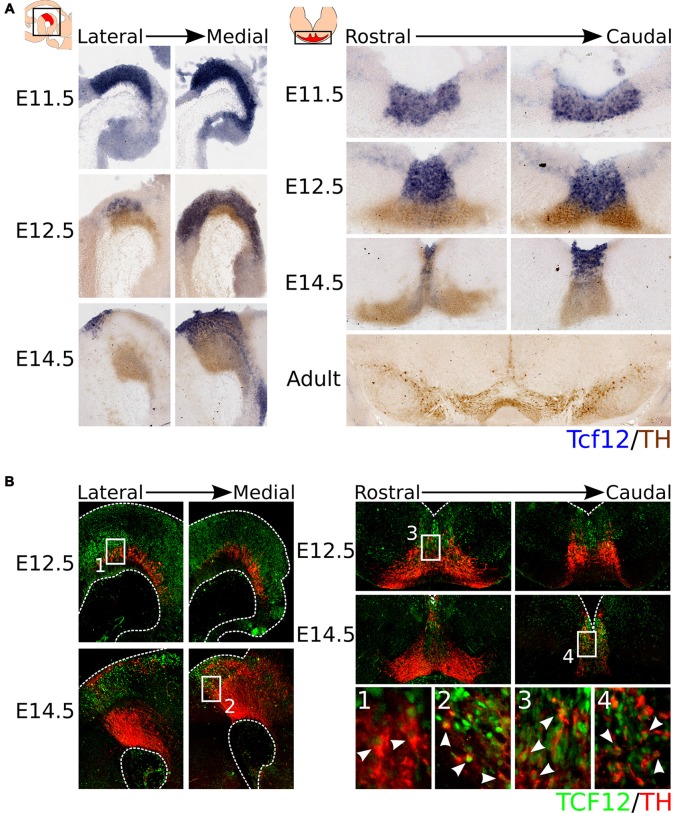
*Tcf12* is expressed throughout development in the midbrain and overlaps with the medial TH-expressing population, reflected in TCF12 protein expression. **(A)** *In situ* hybridization of *Tcf12* (blue) combined with immunohistochemistry for TH (brown) throughout development and in the adult midbrain. *Tcf12* is expressed in the embryonic midbrain from E11.5 until E14.5, but expression is not detected in adult mesodiencephalic dopaminergic (mdDA) neurons. **(B)** Combined immunohistochemistry for TCF12 (green) and TH (red) in E12.5 and E14.5 midbrain. TCF12 protein expression is similar to *Tcf12* transcript expression at E12.5 and E14.5. Expression of TCF12 co-localizes with medially located TH-expressing neurons at E12.5 (**1,3**, white arrowheads), which becomes further restricted to the most caudal and medial parts of the midbrain at E14.5 (**2,4**, white arrowheads).

As introduced above, *Tcf12* is a member of the E-box protein family, a sub-family of the bHLH protein family. The bHLH protein family-members *Ngn2* and *Mash1* have been implicated in the development of mdDA neurons (Andersson et al., 2006a; Kele et al., 2006). To determine whether *Tcf12* is expressed in the midbrain during mdDA development, we characterized its expression pattern by means of *in situ* hybridization (Figure 1A).* Tcf12* is expressed in the VZ of the midbrain and shows a strong expression in the FP at E11.5 and E12.5. At E12.5, *Tcf12* expression overlaps with the TH-expressing population in the most medial sections of the midbrain. At E14.5 the expression of *Tcf12* becomes further restricted and, similarly to E12.5, overlaps with the most medial part of the TH-expressing population. In adult mdDA neurons *Tcf12* transcript could not longer be detected.

In order to confirm the expression pattern of *Tcf12*, we performed immunohistochemistry for TCF12 protein in combination with TH at E12.5 and E14.5. TCF12 protein is present in a similar pattern as *Tcf12* transcript and in concordance co-localizes with part of the medial TH-expressing population (Figure 1B). At E12.5, TCF12 is present in the VZ of the midbrain, and is highly expressed in the FP. Co-localization with TH is detected in both caudally and rostrally (Figures 1B1,3 respectively, white arrowheads) located TH^+^ neurons in the medial part of the midbrain. At E14.5 the expression of TCF12 becomes even more restricted to the medial part of the midbrain and shows co-localization with caudally localized TH^+^ neurons (Figures 1B2,4, white arrowheads).

Together, these data show that *Tcf12* transcript and TCF12 protein are present within the midbrain region and co-localize with part of the TH-expressing neuronal population at E12.5 and E14.5. TH^+^/TCF12^+^ neurons are mostly located in the caudal-medial part of the midbrain area. Since TH-expressing neurons are thought to arise from the FP and BP from E10 onwards and then migrate to more lateral and rostral parts of the midbrain (Ono et al., 2007; Bye et al., 2012; Mesman et al., 2014), the expression pattern of TCF12 suggests that co-localization with TH only occurs in relative young TH^+^ neurons and that *Tcf12* may serve a function in the initial phases of differentiation of neural progenitors towards the mdDA neuronal phenotype.

### Early Loss of *Tcf12* Affects the Expression of the Early mdDA Neuronal Specification Factors *Lmx1a* and *Nurr1*

**Figure 2:**
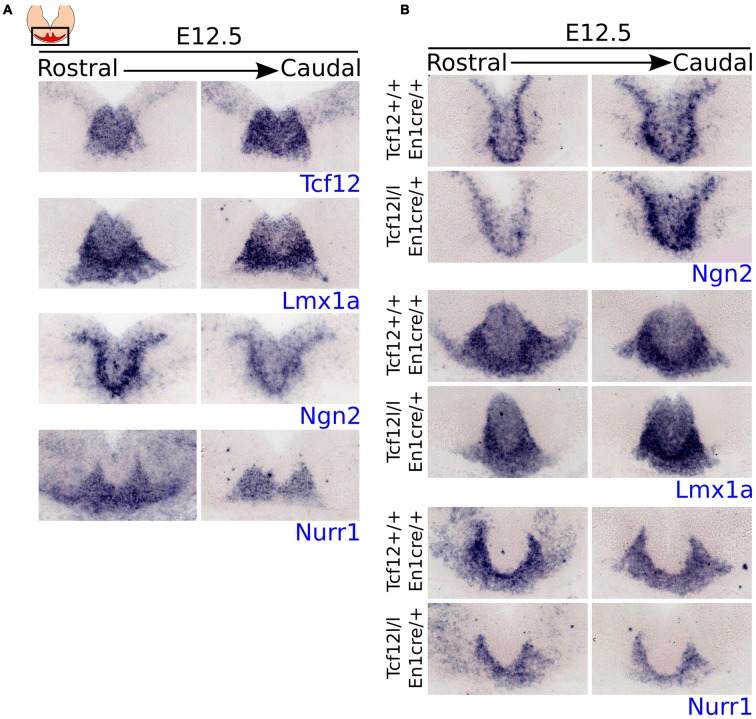
*Tcf12* expression overlaps with the expression of early specification factors in the midbrain and *En1*^cre^ driven deletion of *Tcf12* affects the expression of *Lmx1a* and *Nurr1* but not of *Ngn2*. **(A)** *In situ* hybridization of *Tcf12* (blue), *Lmx1a* (blue), *Ngn2* (blue) and *Nurr1* (blue) at E12.5 in the midbrain.* Tcf12* expression overlaps almost completely with the expression of *Lmx1a* and partially with *Ngn2*. The expression of *Nurr1* overlaps only slightly in the medial parts with *Tcf12*. **(B)** *In situ* hybridization of *Ngn2* (blue), *Lmx1a* (blue) and *Nurr1* (blue) in *Tcf12^lox/lox^*;En1^cre/+^ mutants at E12.5. *En1*^cre^ driven loss of *Tcf12* does not affect the expression pattern of *Ngn2*, but spatially decreases the expression area of *Lmx1a* and *Nurr1*.

As shown above, expression of *Tcf12* is mainly present in the VZ and shows co-localization with relative young TH^+^ neurons, indicating that it coincides with the area in the midbrain that generates mdDA neurons. To confirm that *Tcf12* could serve an early function in mdDA neuronal development, we examined whether the expression of *Tcf12* resembles the expression of known early specification factors in the midbrain (Figure 2A).

Expression of *Tcf12* overlaps almost completely with the expression pattern of *Lmx1a* and partly with *Ngn2*, both important in the early specification of mdDA neurons (Andersson et al., 2006a; Kele et al., 2006; Yan et al., 2011), at E12.5 (adjacent sections). Both factors show a slightly larger area of expression than *Tcf12*, mainly in the ventral region of the expression area of *Tcf12*. *Nurr1*, an important intrinsic differentiation factor of mdDA neurons (Saucedo-Cardenas et al., 1998; Andersson et al., 2007; Kim et al., 2007), on the other hand, overlaps only minimally at the medial parts of *Tcf12* expression at E12.5 (adjacent sections). This expression pattern supports the idea that *Tcf12* is mainly involved in relative early events of the development of mdDA neurons.

The expression pattern of *Tcf12* combined with the expression patterns of early specification factors strongly suggests a role for *Tcf12* in relative early development of mdDA neurons. In order to examine the effect of early loss of *Tcf12* on the expression of these factors we crossed the *Tcf12*^flox^ with the *En1*^cre^ driver (*Tcf12^lox/lox^*;*En1^cre/+^*). *En1* is broadly expressed in the midbrain and R1 from ~E9 onwards (Wurst et al., 1994; Kouwenhoven et al., 2016), resulting in a broad and relative early loss of *Tcf12*. *En1*^cre^ driven loss of *Tcf12* does not display a difference in the expression of *Ngn2* in the midbrain area at E12.5 (Figure 2B, upper panel), whereas in adjacent sections we detected a spatial difference in the expression pattern of both *Lmx1a* (Figure 2B, middle panel) and *Nurr1* (Figure 2B, lower panel). This effect was seen in at least 2 different litters. The *Lmx1a* expression area is smaller in the midbrain of *Tcf12^lox/lox^*;*En1^cre/+^* embryos compared to *Tcf12^+/+^*;*En1^cre/+^* embryos and lacks lateral expression (Figure 2B, middle panel). The expression of *Nurr1* shows a decrease in the area of expression, and similar to the expression of *Lmx1a*, the main effect on the expression of *Nurr1* can be detected in the lateral parts (Figure 2B, lower panel). Together, these data suggest that *Tcf12* influences the expression of other essential early specification factors in mdDA neuronal development.

### *En1^cre^* Driven Loss of *Tcf12* Results in a Delay of mdDA Neuronal Development

**Figure 3:**
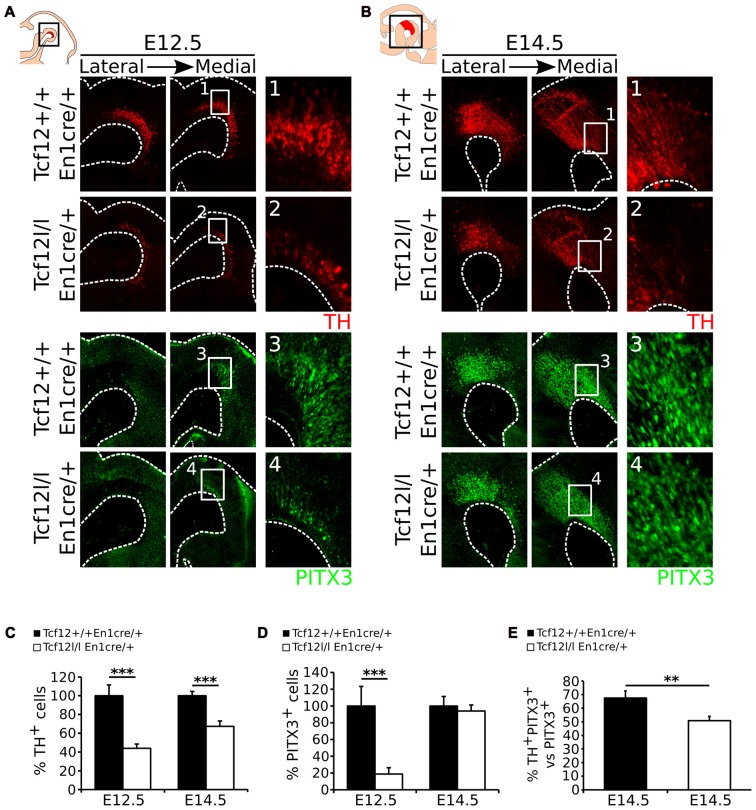
*En1*^cre^ driven loss of *Tcf12* results in a delay in mdDA neuronal cell-fate specification. **(A)** Immunohistochemistry for TH (red) **(1,2)** and PITX3 (green) **(3,4)** in *Tcf12^lox/lox^*;*En1^cre/+^* E12.5 embryos shows a decrease of both markers. **(B)** Immunohistochemistry for TH (red) and PITX3 (green) in *Tcf12^lox/lox^*;*En1^cre/+^* E14.5 embryos shows a rostral decrease in TH expression **(1,2)**, although the population of PITX3^+^ neurons is recovered **(3,4)**. **(C)** Quantification of the TH-expressing cell population at E12.5 and E14.5. The amount of TH-expressing cells is decreased with ~66% at E12.5 (*n* = 3; ***p < 0.001, one-tailed), which is recuperated to a ~33% decrease at E14.5 (*n* = 3; ***p < 0.001 one-tailed) in *Tcf12^lox/lox^*;*En1^cre/+^* (white bars) compared to *Tcf12^+/+^*;*En1^cre/+^* embryos (black bars). *Tcf12^+/+^*;*En1^cre/+^* was set at 100%. **(D)** Quantification of the PITX3-expressing cell population at E12.5 and E14.5. The amount of PITX3-expressing cells is decreased with ~82% at E12.5 (*n* = 3; ***p < 0.001, one-tailed), which is completely recovered at E14.5 in the *Tcf12^lox/lox^*;*En1^cre/+^* (white bars) compared to *Tcf12^+/+^*;*En1^cre/+^* embryos (black bars). *Tcf12^+/+^*;*En1^cre/+^* was set at 100%. **(E)** Quantification of TH^+^/PITX3^+^ cells compared to the total population of PITX3^+^ cells. The amount of TH^+^/PITX3^+^ cells is significantly decreased in *Tcf12*^lox/lox^;*En1*^cre/+^ embryos at E14.5. In *Tcf12*^+/+^;*En1*^cre/+^ embryos (black bar) the amount of TH^+^/PITX3^+^ neurons comprises ~68% of the total PITX3-expressing population, whereas in *Tcf12*^lox/lox^;*En1*^cre/+^ embryos (white bar) this is decreased to ~50% (*n* = 3; **p < 0.01, one-tailed).

**Figure 4:**
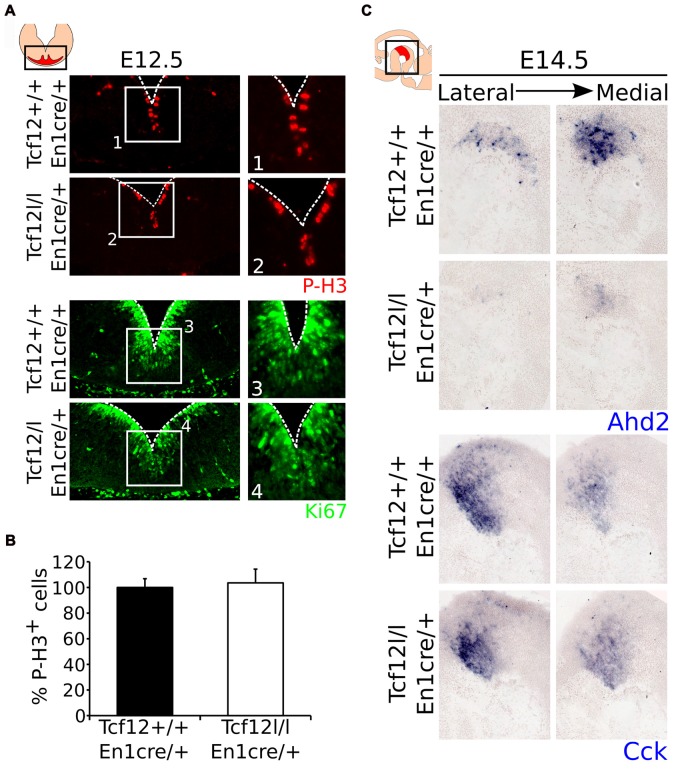
Proliferation is not affected in the ventricular zone (VZ) of the *En1^cre^* driven *Tcf12* mutant and subset specification is mainly affected in the rostral mdDA population. **(A)** Immunohistochemistry of the proliferation markers P-H3 (red) and Ki67 (green) in the murine midbrain at E12.5 shows no apparent difference between *Tcf12*^lox/lox^;*En1*^cre/+^ and *Tcf12*^+/+^;*En1*^cre/+^ embryos (*n* = 3). **(B)** Quantification of the P-H3 expressing cells in the embryonic midbrain at E12.5 shows no difference between the *Tcf12*^lox/lox^;*En1*^cre/+^ (white bar) and the *Tcf12*^+/+^;*En1*^cre/+^ (black bar) embryos. *Tcf12*^+/+^;*En1*^cre/+^ was set at 100%. **(C)** *In situ* hybridization of* Ahd2* and *Cck* in the mutant midbrain at E14.5. Expression of the subset marker *Ahd2* (blue, upper panel) is heavily affected, whereas expression of *Cck* (blue, lower panel) is relatively unaffected in *Tcf12^lox/lox^*;*En1^cre/+^* embryos.

Above we have shown that *En1*^cre^ driven deletion of *Tcf12* results in a spatial difference in expression of *Lmx1a* and *Nurr1*, which are both essential determinants of mdDA neuronal differentiation and subset specification (Saucedo-Cardenas et al., 1998; Andersson et al., 2006b, 2007; Kim et al., 2007; Hoekstra et al., 2013). Since *Nurr1* is expressed in mdDA neurons and deletion of *Nurr1* results in a complete loss of TH-expression in mdDA neurons (Saucedo-Cardenas et al., 1998; Smits et al., 2003), we examined the effect of the loss of *Tcf12* on the appearance of mdDA neurons in the *Tcf12*^lox/lox^;*En1*^cre/+^ mutant at E12.5 and E14.5, by examining the spatio-temporal protein levels of the mdDA neuronal markers TH and PITX3. At E12.5 the presence of TH (Figures 3A1,2) and PITX3 (Figures 3A3,4) is decreased with ~66% (*n* = 3; *p* < 0.001, one-tailed *t*-test; Figure 3C E12.5) and ~82% (*n* = 3; *p* < 0.001, one-tailed *t*-test; Figure 3D E12.5; Absolute numbers are represented in Supplementary Figures S2A–D), respectively. At E14.5 the loss of TH-expressing neurons has recuperated (Figure 3B) to a ~33% decrease (*n* = 3; *p* < 0.001, one-tailed *t*-test; Figure 3C E14.5), whereas the loss of PITX3^+^ neurons is recovered at E14.5 (*n* = 3; Figures 3B,3,4,D, E14.5). The decrease in TH^+^ neurons at E14.5 can mainly be detected in the rostral parts of the TH-expressing population (Figures 3B,1,2). Double-labeling of TH and PITX3 at E14.5 shows that the amount of TH^+^/PITX3^+^ neurons in the total PITX3-expressing population is ~68% in *Tcf12*^+/+^;*En1*^cre/+^ embryos and has decreased to ~50% in the *Tcf12*^lox/lox^;*En1*^cre/+^ embryos (*n* = 3; *p* < 0.01 one-tailed *t-test*; Figure 3E), showing that the population TH^+^/PITX3^+^ neurons has decreased in the mutant. This effect is most prominent in the rostral population of PITX3-expressing neurons (Supplementary Figure S3).

Together, these data show that deletion of *Tcf12* in the *En1* expression domain results in a delay of expression of the mdDA markers TH and PITX3, which partly (TH) or even completely (PITX3) recovers during development.

### *Tcf12* Does Not Influence the Proliferative Capacity of the Midbrain Region

Since *Tcf12* is known to be important in regulation of proliferation and differentiation of T-cells in the immune system and possibly cortical neurons (Uittenbogaard and Chiaramello, 2002; Wojciechowski et al., 2007) and we detected a delay in mdDA neuronal specification, we examined the effect on *En1*^cre^ driven loss of *Tcf12* on the proliferation of neural progenitors in the VZ. MdDA neurons are born from E10 onwards, with neuronal birth peaking at E11-E12 (Bayer et al., 1995; Bye et al., 2012). If the apparent delay in mdDA neuronal differentiation is the result in a delay of proliferation, we would expect that the level of cells that are positive for PhosphoH3 (P-H3) and/or Ki67, both markers for proliferation, would be increased in the mutant animals at E12.5. However, we were not able to detect a clear increase in either P-H3 (Figures 4A1,2,B, Supplementary Figure S2E) or Ki67 (Figures 4A3,4) in the midbrain area between the *Tcf12*^lox/lox^;*En1*^cre/+^ mutant and *Tcf12*^+/+^;*En1*^cre/+^ embryos at E12.5, suggesting that the effects found on TH and PITX3 protein are not a consequence of large changes in proliferation.

### *Tcf12* Influences the Differentiation Program of mdDA Neurons

**Figure 5:**
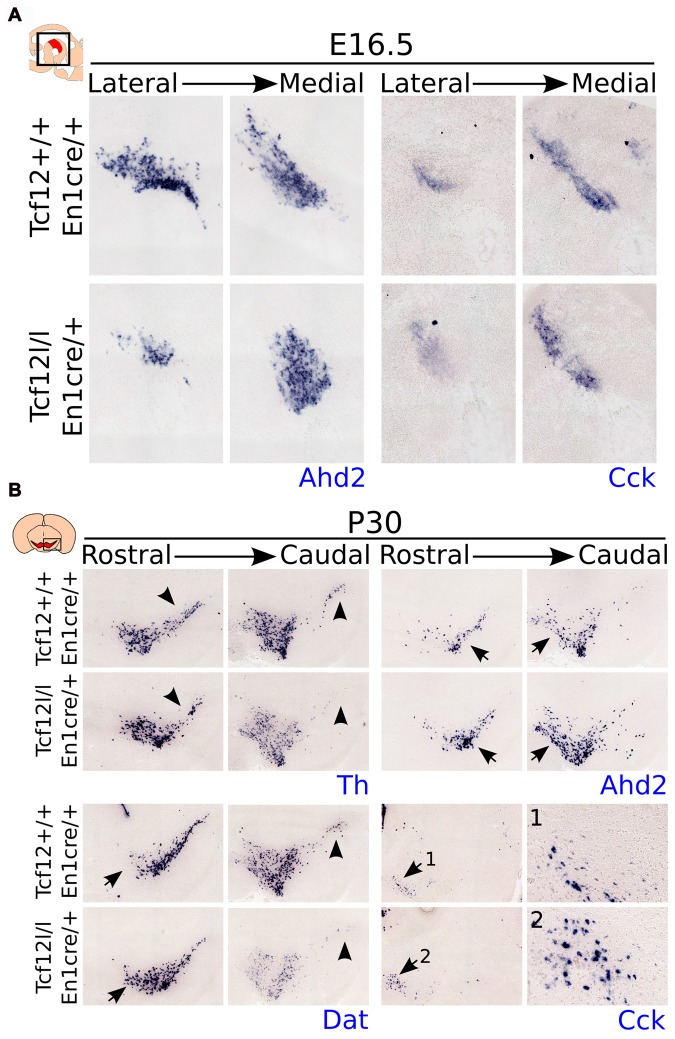
Expression of *Ahd2* remains affected during development in the *Tcf12^lox/lox^*;*En1^cre/+^* mutant midbrain. In the adult, *Th* and *Dat* show a decrease in expression, whereas *Ahd2* and *Cck* expression appears to be increased. **(A)** *In situ* hybridization of *Ahd2* (blue, left panel) and *Cck* (blue, right panel) at E16.5 in the mutant midbrain. *Ahd2* expression is lost in the lateral parts of the midbrain, whereas medially its expression appears to be increased. Expression of *Cck* remains unaffected at E16.5. **(B)** *In situ* hybridization of *Th* (blue, upper left panel)*, Dat* (blue, lower left panel)*, Ahd2* (blue, upper right panel) and *Cck* (blue, lower right panel) in the adult midbrain at P30. Expression of *Th* and *Dat* is decreased in the substantia nigra (SNc) of the adult midbrain (black arrowheads), although *Dat* expression appears to be increased in rostral-medial areas (black arrows), and an increase in expression is detected of *Ahd2* and *Cck* **(1,2)** in caudal/medial parts of the midbrain (black arrows).

As described above, we detected a delay in the development of mdDA neurons unrelated to proliferation defects. This suggests that the differentiation of mdDA neurons is affected and the underlying genetic programming may be altered as well. Since rostral TH expression is still affected at E14.5, whereas the presence of PITX3 is restored, we examined the expression of *Ahd2*, a marker for the rostral mdDA neuronal population and involved in RA dependent *Th* gene activation (Jacobs et al., 2007, 2011), and *Cck*, a marker for the caudal mdDA neuronal population (Veenvliet et al., 2013), in order to validate the programming of emerging mdDA neurons in these mutants. Expression of *Ahd2* is strongly decreased (Figure 4C, upper panel), whereas the expression of *Cck* is relatively unaffected at E14.5 in these mutants (Figure 4C, lower panel), suggesting that rostral mdDA neurons do not only lose expression of TH, but also do not express *Ahd2* at this stage. Taken together, the presented data suggest that *Tcf12* causes a delay in programming and affects the rostral subgroup by an absence of *Ahd2* expression at E14.5. To further substantiate the initial programming defects observed at E14.5 we examined the expression of *Ahd2* and *Cck* at E16.5 and of *Th, Dat, Ahd2*, and *Cck* at P30 (Figure 5). At E16.5 expression of *Ahd2* (Figure 5A, left panel) is partially recovered compared to its expression at E14.5. However, the expression pattern is still affected compared to controls. Similar as described for E14.5, *Cck* is normally expressed in the E16.5 embryonic midbrain (Figure 5A, right panel).

In the adult midbrain expression of the mdDA neuronal markers *Th* (Figure 5B, upper left panel) and *Dat* (Figure 5B, lower left panel) show a modest loss in expression in the lateral and caudal parts of the SNc (Figure 5B, black arrowheads). Interestingly, besides a loss of *Dat* and *Th* expression, the overall level of these markers appears to be affected in caudal parts of the midbrain, whereas in more medial parts of the SNc, an increase in *Dat* transcript level can be detected (black arrows). Expression of the subset markers *Ahd2* (Figure 5B, upper right panel) and *Cck* (Figure 5B, lower right panel 1,2) appears to be increased in adjacent sections (Figure 5B, black arrows). The increase in expression of these subset markers is mainly detected in medial parts of the midbrain. These changes in transcript expression were seen in several adult animals.

**Figure 6:**
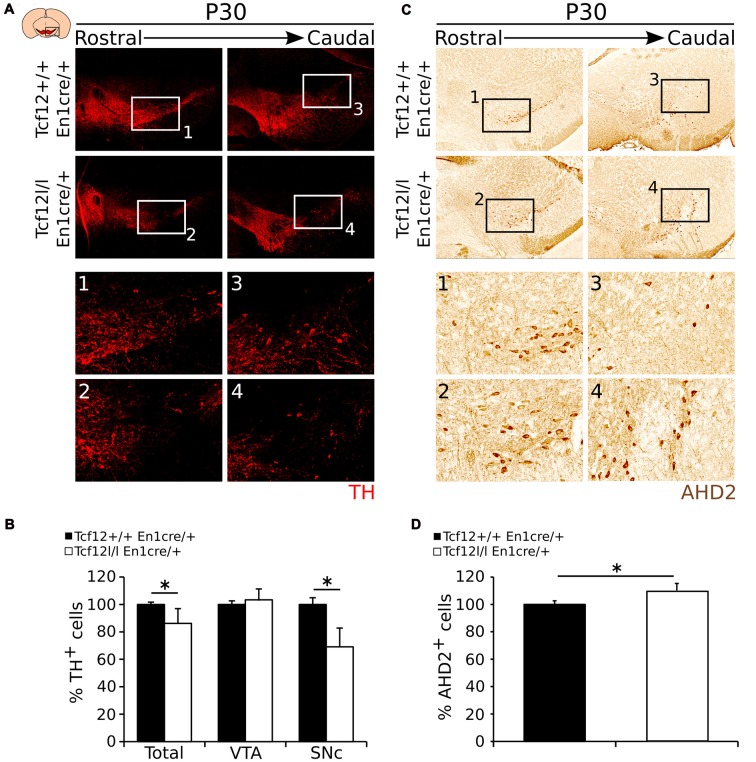
TH shows a decrease in expression in the adult *Tcf12^lox/lox^*;*En1^cre/+^* mutant midbrain, whereas AHD2 expression is increased with ~10%. **(A)** Immunohistochemistry for TH (red) in the adult midbrain at P30. TH^+^ cells are lost in the SNc of the mutant midbrain at P30 compared to the WT **(1–4)**. **(B)** Quantification of the number of TH^+^ cells in *Tcf12^lox/lox^*;*En1^cre/+^* (white bars) compared to *Tcf12^+/+^*;*En1^cre/+^* (black bars) midbrain at P30. The total amount of TH-expressing cells is decreased with ~14% in the midbrain (*n* = 3; *p < 0.05, one-tailed), which is represented in the SNc with a ~31% decreased (*n* = 3; *p < 0.05, one-tailed), but not in the ventral tegmental area (VTA; *n* = 3). *Tcf12^+/+^*;*En1^cre/+^* was set at 100%. **(C)** Immunohistochemistry for AHD2 (brown) in the adult midbrain at P30. AHD2^+^ neurons show an increase in the mutant midbrain at P30 compared to the WT **(1–4)**. **(D)** Quantification of the number of AHD2^+^ cells in the *Tcf12^lox/lox^*;*En1^cre/+^* (white bar) compared to *Tcf12^+/+^*;*En1^cre/+^* (black bar) midbrain at P30. The total amount of AHD2-expressing cells is increased with ~10% in the midbrain (*n* = 3; *p < 0.05, one-tailed). *Tcf12^+/+^*;*En1^cre/+^* was set at 100%.

To substantiate the expression data as described above, we aimed to quantify the amount of TH- and AHD2-expressing neurons in the adult mdDA neuronal population. In accordance to *Th* transcript, TH-expressing cells are specifically lost in the SNc region (Figures 6A1–4). The total amount of TH-expressing cells is ~14% lower in *Tcf12^lox/lox^*;*En1^cre/+^* (*n* = 3; *p* < 0.05, one-tailed) than in *Tcf12^+/+^*;*En1^cre/+^* littermates (Figure 6B, Supplementary Figure S2F). The reduction in TH-expressing cells is only observed in the SNc where the amount of TH^+^ neurons is reduced with ~31% (*n* = 3; *p* < 0.05, one tailed), whereas in the VTA the amount of TH^+^ neurons is not significantly changed in *Tcf12^lox/lox^*;*En1^cre/+^* animals (*n* = 3; Figure 6B). Similar to the increased *Ahd2* transcript expression area, an increase in AHD2-expressing cells (Figures 6C1–4) of ~10% (*n* = 3; *p* < 0.05, one-tailed) in *Tcf12^lox/lox^*; *En1^cre/+^* animals (Figure 6D, Supplementary Figure S2G) is detected. Together, these data show that the population of TH-expressing neurons is specifically affected in the SNc in the adult stage. The observed increase in AHD2-containing neurons within the mdDA neuronal population suggests that the subset programming of the remaining mdDA neurons in the mutant is affected as well.

### *Pitx3^cre^* Driven Ablation of *Tcf12* Influences Programming of mdDA Neurons

Above we have shown that early deletion of *Tcf12* under the *En1*^cre^ driver results in a loss of TH expression in the SNc and overall programming defects. The latter suggests that *Tcf12* may influence terminal differentiation of mdDA neurons. In order to validate this notion we created a *Pitx3^cre^* driven conditional ablation of *Tcf12*.

**Figure 7:**
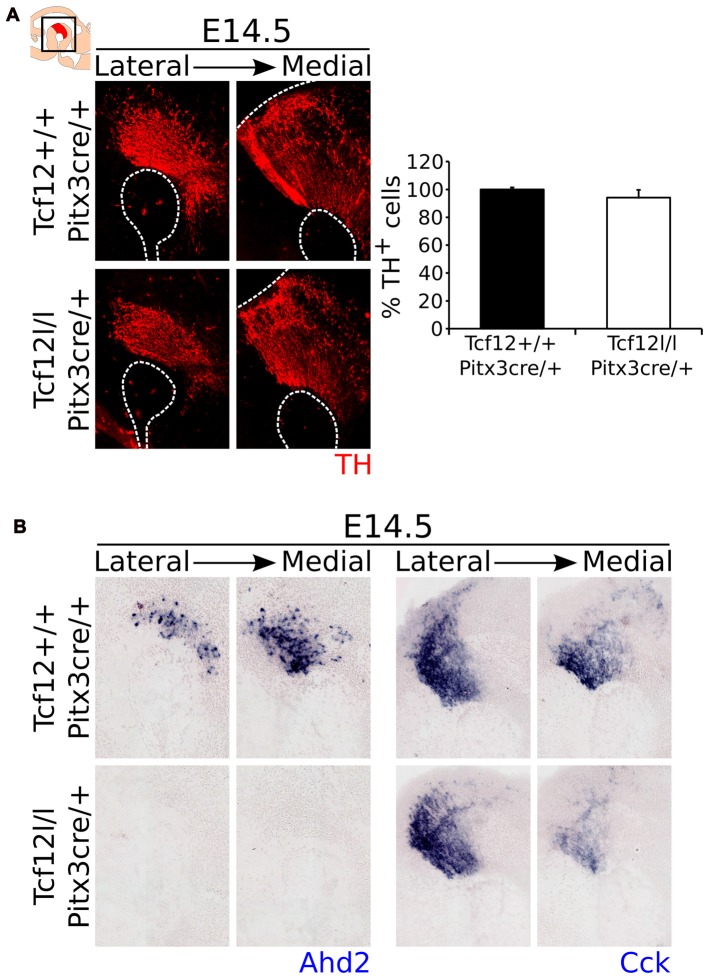
*Pitx3*^cre^ driven loss of *Tcf12* has no effect on the amount of TH-expressing cells, but results in a delay in subset specification at E14.5. **(A)** Immunohistochemistry for TH (red) in *Tcf12^lox/lox^*;*Pitx3^cre/+^* (white bar) and *Tcf12^+/+^*;*Pitx3^cre/+^* (black bar) embryos at E14.5 shows no difference in the distribution or amount of TH-expressing cells (*n* = 3). *Tcf12^+/+^*;*Pitx3^cre/+^* was set at 100%. **(B)** *In situ* hybridization of the subset markers *Ahd2* (blue, left panel) and *Cck* (blue, right panel) in the mutant midbrain at E14.5. Expression of *Ahd2* is completely lost, whereas *Cck* appears to be only affected in the most medial sections of the midbrain in *Tcf12^lox/lox^*;*Pitx3^cre/+^* embryos.

Contrary to a relative early deletion of *Tcf12*, *Pitx3*^cre^ driven deletion of *Tcf12* does not affect TH expression in mdDA neurons at E14.5 (Figure 7A, Supplementary Figure S2H, *n* = 3). Interestingly, expression of the subset marker *Ahd2* is similarly lost in the *Tcf12^lox/lox^*;*Pitx3^cre/+^* mutant at E14.5 (Figure 7B, left panel), whereas the spatial expression of *Cck* appears to be diminished in the rostral-medial expression area (Figure 7B, right panel). These data corroborate the initial finding that *Tcf12* does influence the programming of mdDA neurons in the terminal differentiation state.

**Figure 8:**
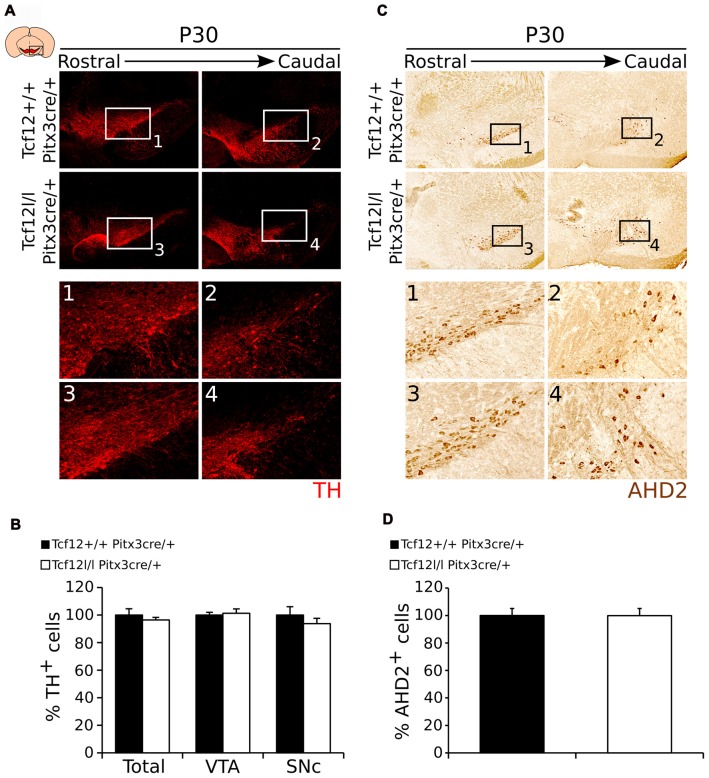
The mdDA neuronal population is normally present in the adult midbrain of Tcf12^lox/lox^;Pitx3^cre/+^ animals. **(A)** Immunohistochemistry of TH (red) in the midbrain of *Tcf12^lox/lox^*;*Pitx3^cre/+^* and *Tcf12^+/+^*;*Pitx3^cre/+^* animals. Expression of TH is not apparently affected in the mutant midbrain at P30 **(1–4)**. **(B)** Quantification of TH in *Tcf12^lox/lox^*;*Pitx3^cre/+^* (white bars) compared to *Tcf12^+/+^*;*Pitx3^cre/+^* (black bars) animals at P30. The total amount and distribution between the SNc and VTA of TH-expressing neurons in the adult midbrain is not changed between the mutant and the WT animals (*n* = 3). *Tcf12^+/+^*;*Pitx3^cre/+^* was set at 100%. **(C)** Immunohistochemistry of AHD2 (brown) in the midbrain of *Tcf12^lox/lox^*;*Pitx3^cre/+^* and *Tcf12^+/+^*;*Pitx3^cre/+^* animals. Expression of AHD2 is not apparently affected in the mutant midbrain at P30 **(1–4)**. **(D)** Quantification of AHD2 in *Tcf12^lox/lox^*;*Pitx3^cre/+^* (white bar) compared to *Tcf12^+/+^*;*Pitx3^cre/+^* (black bar) animals at P30 shows no effect of the loss of *Tcf12* in amount of AHD2^+^ neurons (*n* = 3). *Tcf12^+/+^*;*Pitx3^cre/+^* was set at 100%.

In order to follow the development of the system and confirm these programming defects in adult mutants, we quantified the amount of TH- (Figure 8A) and AHD2-expressing cells (Figure 8C). Quantification of the total amount of TH-expressing neurons and in the SNc and VTA separately, shows, in concordance to earlier time-points, that the amount of TH neurons is unaffected by late ablation of *Tcf12* (Figures 8A1–4,B, Supplementary Figure S2I, *n* = 3). Noteworthy, although *Ahd2* expression is completely lost in *Tcf12^lox/lox^*;*Pitx3^cre/+^* embryos at E14.5, this effect is absent at P30 (Figures 8C1–4,D, Supplementary Figure S2J, *n* = 3). Not only could this be detected at the protein level, also transcript expression of *Th, Ahd2* and *Cck* in the adult midbrain was unaffected in *Tcf12^lox/lox^*;*Pitx3^cre/+^* animals (Supplementary Figure S4).

Taken together these data show that *Pitx3*^cre^ driven deletion of *Tcf12* confirms the temporal delay in correct coding of mdDA neurons, which, in late ablation of *Tcf12*, is corrected in the adult system.

## Discussion

The mdDA neuronal population develops from the VZ in the mesodiencephalic FP and BP from E10 onwards (Bayer et al., 1995). During this development proneural bHLH factors, like *Ngn2* and *Mash1*, have been shown to be important in the development of the mdDA neuronal population (Andersson et al., 2006a; Kele et al., 2006). However, although many other bHLH factors are known to be expressed in the midbrain area, few studies have been performed to elucidate the possible role of these factors during mdDA neuronal differentiation. In this study, we have established the E-box protein *Tcf12*, which is part of the bHLH sub-family the E-box proteins, as an important factor both in early mdDA neuronal cell-fate commitment and in late subset formation.

Here, we have shown that *Tcf12* transcript and protein are expressed in the VZ of the midbrain and partly co-localize with newly generated TH^+^ neurons and early mdDA specification factors like *Lmx1a, Nurr1* and *Ngn2*. *En1*^cre^ driven deletion of *Tcf12* leads to a change in the spatial expression of *Lmx1a* and *Nurr1*, but not of *Ngn2*. Interestingly, *Tcf12* expression slightly overlaps with the expression of *Nurr1* in the midbrain, indicating that the effect on the *Nurr1-*expressing population is established earlier during development.

Besides its effect on the expression of early specification factors, *Tcf12*^lox/lox^;*En1*^cre/+^ embryos show a strong delay in the expression of the mdDA markers TH and PITX3. However, although PITX3 expression completely recovers during development, the rostral population of TH-expressing neurons remains affected during development until the adult stage. This delay in mdDA marker expression was not detected in *Tcf12*^lox/lox^;*Pitx3*^cre/+^ embryos, indicating that it is likely caused by a function of *Tcf12* early in the specification of neural progenitor cells (NPCs). Interestingly, we did not detect a delay in proliferation, as the proliferation markers P-H3 and Ki67 were not noticeably altered in the mutant. Therefore, loss of *Tcf12* under the *En1*^cre^ driver likely leads to an affected differentiation, in which NPCs are normally initiated to leave their proliferating state and start differentiating, but cell-fate commitment into fully differentiated neurons is affected.

Besides the delay in expression of the mdDA neuronal markers TH and PITX3, *Tcf12*^lox/lox^;*En1*^cre/+^ mutants show a delay in expression of the subset marker *Ahd2*, which eventually results in an increase in AHD2-expressing cells at P30. *Pitx3* has previously been shown to affect the expression of *Ahd2* (Jacobs et al., 2011), indicating that the delay in PITX3 expression could in part be responsible for the delay in expression of *Ahd2*. However, expression of PITX3 was completely recovered at E14.5 in these mutants, which is around the time of initial expression of *Ahd2* (McCaffery and Dräger, 1994). Hence, the initial loss of *Ahd2* expression could not solely be due to the delay in PITX3 expression. Interestingly, the *Tcf12^lox/lox^*;*Pitx3^cre/+^* mutants show a similar delay of *Ahd2* expression, indicating that, besides an early role in mdDA neuronal differentiation, *Tcf12* has a late role in subset specification which is possibly unrelated to *Pitx3* function. This late role of *Tcf12* is likely also responsible for the loss of *Ahd2* expression in *Tcf12^lox/lox^*;*En1^cre/+^* E14.5 embryos.

Although *Ahd2* is not expressed in the same region as *Tcf12*, both conditional approaches showed a specific effect on *Ahd2* expression. These data suggest that regulation of subset specification already starts in young TH^+^ neurons that express *Tcf12* and further develops during neuronal migration. The delay in *Ahd2* expression in *Tcf12^lox/lox^*;*En1^cre/+^* midbrain could be responsible for the loss of the rostral TH-expressing group of neurons, since *Ahd2* has been shown to be important in RA-dependent activation of *Th* in the midbrain (Jacobs et al., 2007, 2011). In combination with the early effects of *Tcf12* deletion, this could lead to a loss of TH-expressing neurons and increase in caudally located AHD2^+^ cells. Interestingly, the loss of TH-expressing neurons in the SNc is similar to what can be detected in the mdDA neuronal population of patients suffering from PD. Since *Tcf12* appears to be important in the correct differentiation of mdDA neurons and subset specification at later stages, this would be a possible candidate gene to study in relation to culturing correct SNc neuron from iPSCs in the use for cell replacement therapies in PD.

The difference in loss of mdDA neuronal markers between the *En1^cre^*-driven deletion and the *Pitx3^cre^*-driven deletion of *Tcf12* may be due to two main differences between these two drivers. First of all there is a timing difference in the loss of *Tcf12* with the use of these two drivers. *En1* is expressed at an earlier stage than *Pitx3* (~E9 and ~E12 respectively), meaning that *Tcf12* is lost at least 2–3 days earlier in the *En1^cre^*-driven deletion than in the *Pitx3^cre^*-driven deletion. Second of all *En1* is expressed in an area partly outside mdDA neurons, and can be detected in the FP at early stages (Wurst et al., 1994; Kouwenhoven et al., 2016), whereas *Pitx3* expression is specific for mdDA neurons (Smidt et al., 2012). Taken together, based on the spatial-temporal difference in *Tcf12* deletion, our data suggests that *Tcf12* is possibly involved early in the FP of the midbrain in correct differentiation of DA progenitors to mdDA neurons, as detected in the *En1^cre^* driven deletion of *Tcf12*, whereas it has a later role within mdDA neurons to ensure the correct and timely subset specification, as detected in the *Pitx3^cre^* driven deletion of *Tcf12*.

This indicates that *Tcf12* is a novel factor in mdDA neuronal development, and has a dual role in correct differentiation of mdDA neurons; an early role in cell-fate commitment and a late role mdDA subset specification.

## Author Contributions

SM and MPS: conceived and designed the experiments; analyzed the data; contributed reagents/materials/analysis tools; wrote the article. SM: performed the experiments.

## Conflict of Interest Statement

The authors declare that the research was conducted in the absence of any commercial or financial relationships that could be construed as a potential conflict of interest.

## Supplementary Material

FIGURE S1 Genotyping of *Pitx3^cre^*, *En1^cre^* and *Tcf12^flox^* alleles.** (A)** Genotyping of the *Tcf12^flox^* allele results in a clear PCR product at 1.1 kb for the WT allele and 1.3 kb for the cre-allele. **(B)** Genotyping of the *En1^cre^* allele results in a clear PCR product at 240 bp for the cre-allele. **(C)** Genotyping of the *Pitx3^cre^* allele results in a clear PCR product at 750 bp for the cre-allele, and a clear PCR product at 400 bp for the WT allele. m: DNA marker; (-): negative control; E1: example 1; E2: example 2; E3: example 3.

FIGURE S2 Absolute quantifications of *Tcf12^flox^* animals. Note that numbers in this figure represent actual counted neurons and therefore give an under representation of the actual number of mesodiencephalic dopaminergic (mdDA) neurons in the mouse midbrain. **(A)** Absolute quantification of the amount of TH^+^ neurons in *Tcf12^+/+^*;En1^cre/+^ (black bars) compared to *Tcf12^lox/lox^*;En1^cre/+^ (white bars) embryos at E12.5 and E14.5. **(B)** Absolute quantification of the amount of PITX3^+^ neurons in *Tcf12^+/+^*;En1^cre/+^ (black bars) compared to *Tcf12^lox/lox^*;En1^cre/+^ (white bars) embryos at E12.5 and E14.5. **(C)** Absolute quantification of the amount of TH^+^ neurons in *Tcf12^+/+^*;En1^cre/+^ and *Tcf12^lox/lox^*;En1^cre/+^ embryos at E12.5 (black bars) compared to E14.5 (white bars). **(D)** Absolute quantification of the amount of PITX3^+^ neurons in *Tcf12^+/+^*;En1^cre/+^ and *Tcf12^lox/lox^*;En1^cre/+^ embryos at E12.5 (black bars) compared to E14.5 (white bars). **(E)** Absolute quantification of the amount of P-H3^+^ cells at E12.5 in *Tcf12^+/+^*;En1^cre/+^ (black bar) compared to *Tcf12^lox/lox^*;En1^cre/+^ white bar) embryos. **(F)** Absolute quantification of the amount of TH^+^ neurons in the ventral tegmental area (VTA), substantia nigra (SNc), and total mdDA population *Tcf12^+/+^*;En1^cre/+^ (black bars) and *Tcf12^lox/lox^*;En1^cre/+^ (white bars) mice at P30. **(G)** Absolute quantification of the amount of AHD2^+^ neurons in *Tcf12^+/+^*;En1^cre/+^ (black bar) and *Tcf12^lox/lox^*;En1^cre/+^ (white bar) mice at P30. **(H)** Absolute quantification of the amount of TH^+^ neurons in *Tcf12^+/+^*;Pitx3^cre/+^ (black bar) and *Tcf12^lox/lox^*;Pitx3^cre/+^ (white bar) embryos at E14.5. **(I)** Absolute quantification of the amount of TH^+^ neurons in the VTA, SNc and total mdDA population *Tcf12^+/+^*;Pitx3^cre/+^ (black bars) and *Tcf12^lox/lox^*;Pitx3^cre/+^ (white bars) mice at P30. **(J)** Absolute quantification of the amount of AHD2^+^ neurons in *Tcf12^+/+^*;Pitx3^cre/+^ (black bar) and *Tcf12^lox/lox^*;Pitx3^cre/+^ (white bar) mice at P30. *p* < 0.05 is represented by a * and *p* < 0.001 is represented by ***.

FIGURE S3 Rostral TH^−^/PITX3^+^ expressing population in the *Tcf12*^lox/lox^;En1^cre/+^ mutant is increased when compared to the WT expression. Co-localization of TH (red) and PITX3 (green) is decreased in *Tcf12^lox/lox^*;*En1^cre/+^* E14.5 embryos, resulting in an increase in the rostral PITX3-only expressing cells in the mdDA system (blow-ups **1,2**, **1′,2′**, and **1″,2″**).

FIGURE S4 Expression of *Th, Ahd2* and *Cck* in the adult mdDA system is unaffected upon *Pitx3^cre^* driven loss of *Tcf12*. Expression of *Th, Ahd2* and *Cck* is unaffected in *Tcf12^lox/lox^*;Pitx3^*cre/+*^ and *Tcf12^+/+^*;Pitx3*^cre/+^* animals in the adult mdDA neuronal population.
