# Supplementary figures and images for: Tcf12 Is Involved in Early Cell-Fate Determination and Subset Specification of Midbrain Dopamine Neurons
Source: Front Mol Neurosci. 2017 Nov 1;10:353. doi: 10.3389/fnmol.2017.00353 (PMC5671939; doi:10.3389/fnmol.2017.00353)

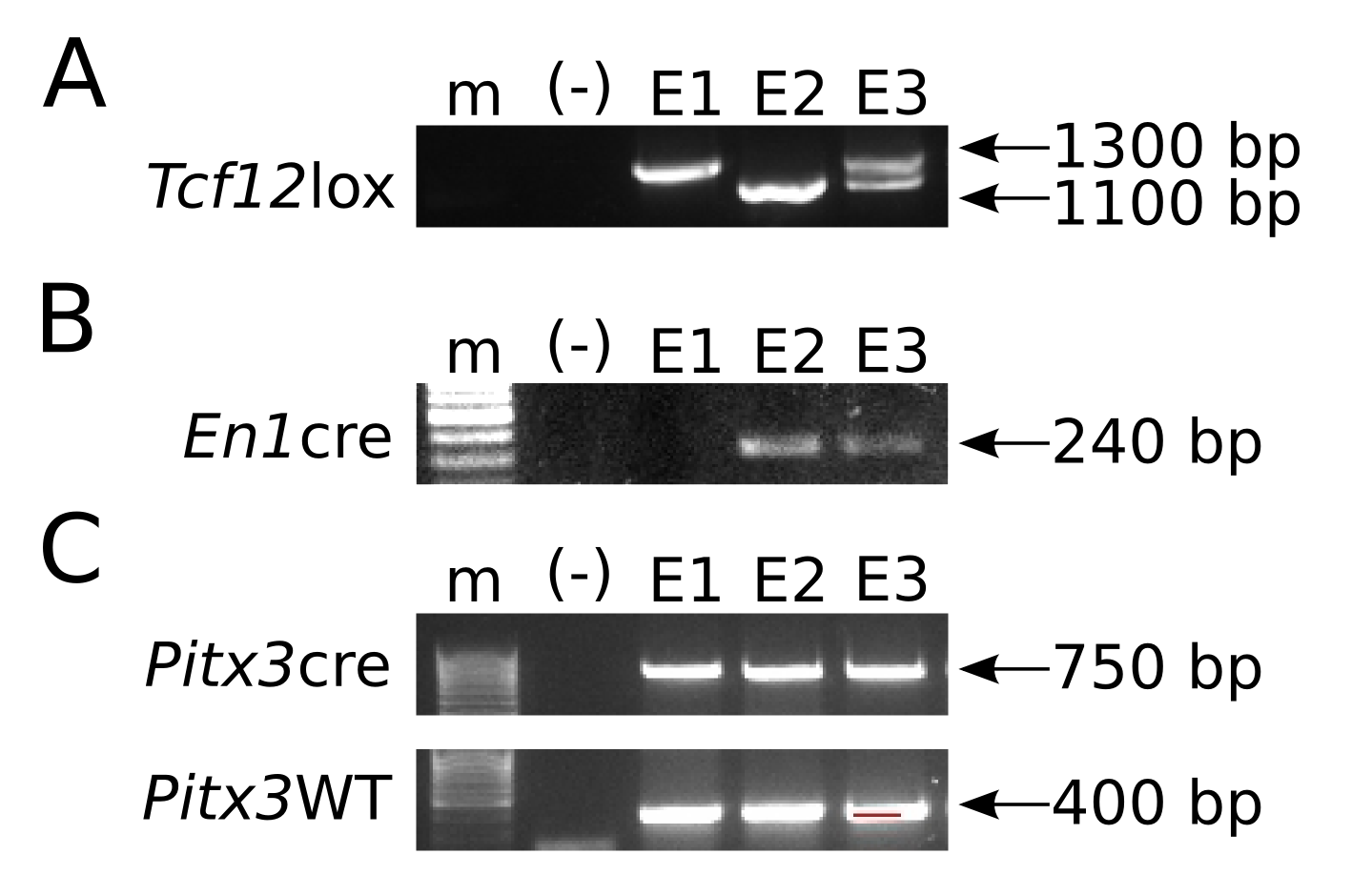

Supplement: FIGURE S1 — Genotyping of Pitx3cre, En1cre and Tcf12flox alleles. (A) Genotyping of the Tcf12flox allele results in a clear PCR product at 1.1 kb for the WT allele and 1.3 kb for the cre-allele. (B) Genotyping of the En1cre allele results in a clear PCR product at 240 bp for the cre-allele. (C) Genotyping of the Pitx3cre allele results in a clear PCR product at 750 bp for the cre-allele, and a clear PCR product at 400 bp for the WT allele. m: DNA marker; (-): negative control; E1: example 1; E2: example 2; E3: example 3. [file Image_1.tif]

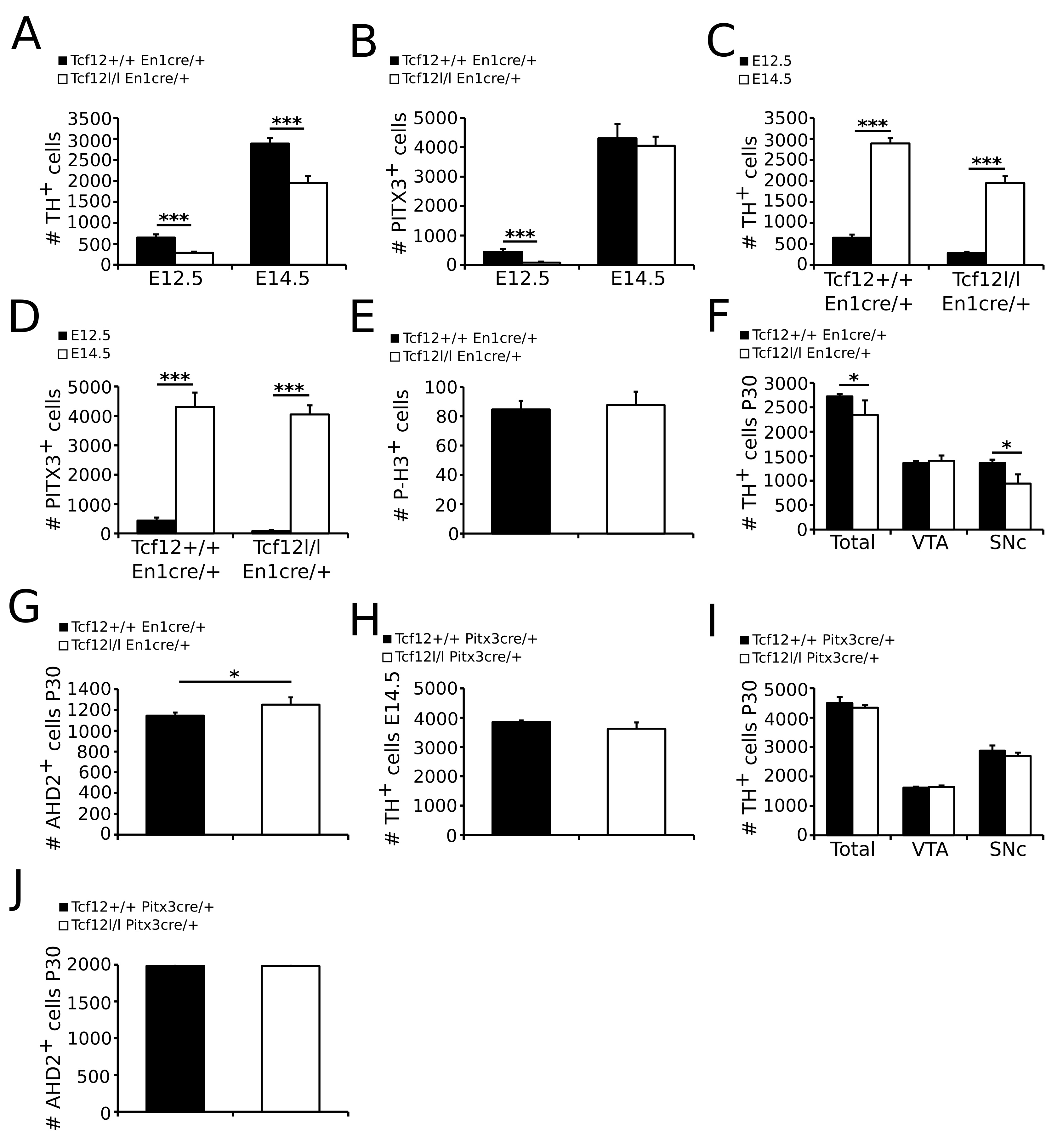

Supplement: FIGURE S2 — Absolute quantifications of Tcf12flox animals. Note that numbers in this figure represent actual counted neurons and therefore give an under representation of the actual number of mesodiencephalic dopaminergic (mdDA) neurons in the mouse midbrain. (A) Absolute quantification of the amount of TH+ neurons in Tcf12+/+;En1cre/+ (black bars) compared to Tcf12lox/lox;En1cre/+ (white bars) embryos at E12.5 and E14.5. (B) Absolute quantification of the amount of PITX3+ neurons in Tcf12+/+;En1cre/+ (black bars) compared to Tcf12lox/lox;En1cre/+ (white bars) embryos at E12.5 and E14.5. (C) Absolute quantification of the amount of TH+ neurons in Tcf12+/+;En1cre/+ and Tcf12lox/lox;En1cre/+ embryos at E12.5 (black bars) compared to E14.5 (white bars). (D) Absolute quantification of the amount of PITX3+ neurons in Tcf12+/+;En1cre/+ and Tcf12lox/lox;En1cre/+ embryos at E12.5 (black bars) compared to E14.5 (white bars). (E) Absolute quantification of the amount of P-H3+ cells at E12.5 in Tcf12+/+;En1cre/+ (black bar) compared to Tcf12lox/lox;En1cre/+ white bar) embryos. (F) Absolute quantification of the amount of TH+ neurons in the ventral tegmental area (VTA), substantia nigra (SNc), and total mdDA population Tcf12+/+;En1cre/+ (black bars) and Tcf12lox/lox;En1cre/+ (white bars) mice at P30. (G) Absolute quantification of the amount of AHD2+ neurons in Tcf12+/+;En1cre/+ (black bar) and Tcf12lox/lox;En1cre/+ (white bar) mice at P30. (H) Absolute quantification of the amount of TH+ neurons in Tcf12+/+;Pitx3cre/+ (black bar) and Tcf12lox/lox;Pitx3cre/+ (white bar) embryos at E14.5. (I) Absolute quantification of the amount of TH+ neurons in the VTA, SNc and total mdDA population Tcf12+/+;Pitx3cre/+ (black bars) and Tcf12lox/lox;Pitx3cre/+ (white bars) mice at P30. (J) Absolute quantification of the amount of AHD2+ neurons in Tcf12+/+;Pitx3cre/+ (black bar) and Tcf12lox/lox;Pitx3cre/+ (white bar) mice at P30. p < 0.05 is represented by a * and p < 0.001 is represented by ***. [file Image_2.tif]

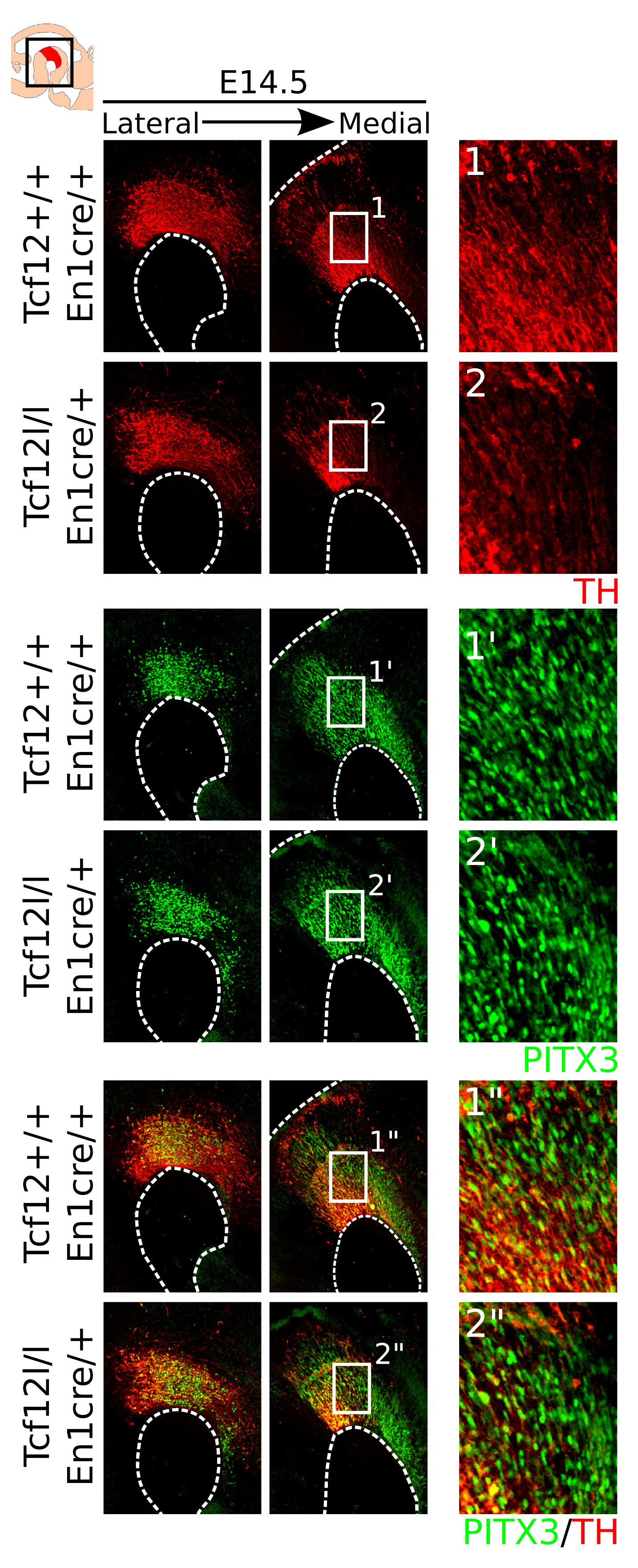

Supplement: FIGURE S3 — Rostral TH−/PITX3+ expressing population in the Tcf12lox/lox;En1cre/+ mutant is increased when compared to the WT expression. Co-localization of TH (red) and PITX3 (green) is decreased in Tcf12lox/lox;En1cre/+ E14.5 embryos, resulting in an increase in the rostral PITX3-only expressing cells in the mdDA system (blow-ups 1,2, 1′,2′, and 1″,2″). [file Image_3.tif]

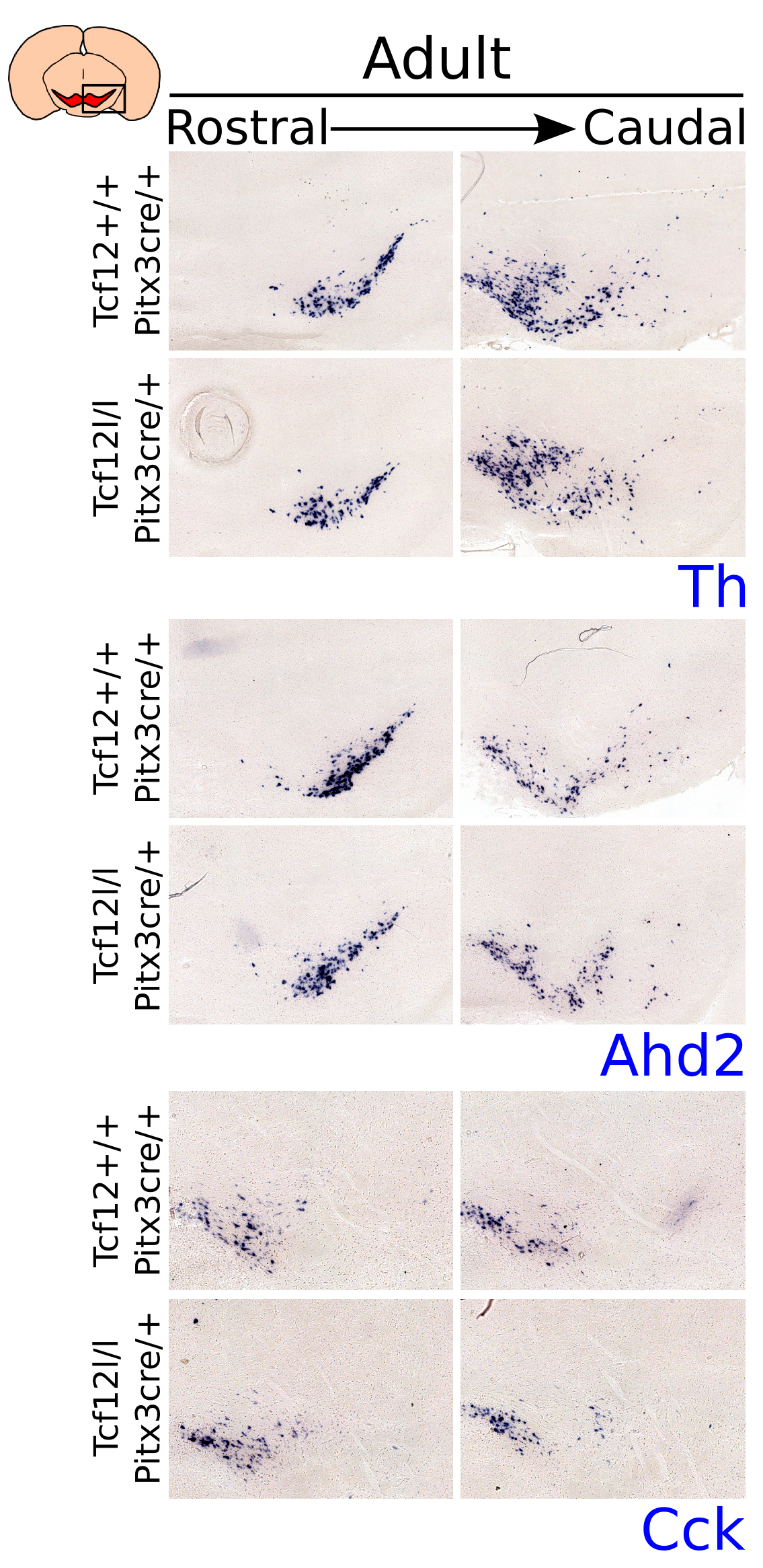

Supplement: FIGURE S4 — Expression of Th, Ahd2 and Cck in the adult mdDA system is unaffected upon Pitx3cre driven loss of Tcf12. Expression of Th, Ahd2 and Cck is unaffected in Tcf12lox/lox;Pitx3cre/+ and Tcf12+/+;Pitx3cre/+ animals in the adult mdDA neuronal population. [file Image_4.tif]
